# Mocetinostat combined with gemcitabine for the treatment of leiomyosarcoma: Preclinical correlates

**DOI:** 10.1371/journal.pone.0188859

**Published:** 2017-11-29

**Authors:** Gonzalo Lopez, Danielle Braggio, Abeba Zewdu, Lucia Casadei, Kara Batte, Hemant Kumar Bid, David Koller, Peter Yu, Obiajulu Hans Iwenofu, Anne Strohecker, Edwin Choy, Dina Lev, Raphael Pollock

**Affiliations:** 1 Comprehensive Cancer Center, The Ohio State University, Columbus, OH, United States of America; 2 Life Science Institute, University of Michigan, Ann Arbor, MI, United States of America; 3 Department of Pathology, The Ohio State University, Columbus, OH, United States of America; 4 Division of Hematology Oncology, Massachusetts General Hospital, Boston, MA, United States of America; 5 Surgery B, Sheba Medical Center, Tel Aviv, Israel; 6 Department of Surgery, The Ohio State University, Columbus, OH, United States of America; Columbia University, UNITED STATES

## Abstract

Leiomyosarcoma (LMS) is a malignant soft tissue sarcoma (STS) with a dismal prognosis following metastatic disease. Chemotherapeutic intervention has demonstrated to have modest clinical efficacy with no curative potential in LMS patients. Previously, we demonstrated pan-HDAC inhibition to have a superior effect in various complex karyotypic sarcomas. In this study, our goal is to evaluate the therapeutic efficacy of mocetinostat alone and in combination with gemcitabine in LMS. Human leiomyosarcoma (LMS) cell lines were used for *in vitro* and *in vivo* studies. Compounds tested included the class I HDAC inhibitor, mocetinostat, and nucleoside analog, gemcitabine. MTS and clonogenic assays were used to evaluate the effect of mocetinostat on LMS cell growth. Cleaved caspase 3/7 analysis was used to determine the effects of mocetinostat on apoptosis. Compusyn software was used to determine *in vitro* synergy studies for the combination of mocetinostat plus gemcitabine. A LMS xenograft model in SCID mice was used to test the impact of mocetinostat alone, gemcitabine alone and the combination of mocetinostat plus gemcitabine. Mocetinostat abrogated LMS cell growth and clonogenic potential, and enhanced apoptosis in LMS cell lines. The combination of mocetinostat plus gemcitabine exhibited a synergistic effect in LMS cells *in vitro*. Similarly, mocetinostat combined with gemcitabine resulted in superior anti-LMS effects *in vivo*. Mocetinostat reduced the expression of gemcitabine-resistance markers RRM1, RRM2, and increased the expression of gemcitabine-sensitivity marker, hENT1, in LMS cells. LMS are aggressive, metastatic tumors with poor prognosis where effective therapeutic interventions are wanting. Our studies demonstrate the potential utility of mocetinostat combined with gemcitabine for the treatment of LMS.

## Introduction

Leiomyosarcoma (LMS) is a malignant tumor of mesenchymal origin, and is one of the most common soft tissue sarcoma subtypes. LMS can present in various locations including the uterus, skin, blood vessels, retroperitoneum, gastrointestinal tract, trunk, and extremities [1]. The pathogenesis of LMS remains largely unknown, with complex karyotype and both intra- and intertumor heterogeneity [2]. Advanced-stage LMS is usually incurable with current systemic therapies, suggesting the need for novel antitumor strategies [1].

A common LMS systemic therapeutic option includes gemcitabine (2’,2’-difluorodeoxycytidine) combined with docetaxel [3]. Gemcitabine is a fluorinated analogue of the nucleoside deoxycytidine. Intracellular phosphorylation of gemcitabine leads to an active diphosphate form which inhibits ribonucleotide reductase and an active triphosphate form which is incorporated into DNA [4]. Administration of gemcitabine alone was reported to have a response rate of 21% in second-line therapy in uterine LMS based on Gynecological Oncology Group (GOG) response criteria [5]. In a phase II study, gemcitabine combined with docetaxel showed a response rate of 25% by RECIST criteria [6]. Even in the first-line setting, a phase II trial of locally advanced/metastatic LMS demonstrated that gemcitabine combined with docetaxel was active [7]. The combination of gemcitabine and docetaxel are synergistic [8], raising the possibility that gemcitabine be synergistic with other anticancer therapeutics as well.

One antitumor strategy of growing interest are histone deacetylases (HDAC) inhibitors [9]. HDACs play a crucial role in chromatin remodeling and gene regulation; histone acetylation of lysines in histone tails is associated with a condensed chromatin state and gene silencing [10]. Inhibition of this process leads to a more relaxed chromatin state and active gene transcription [11]. There are 11 isoforms of HDACs which are grouped into 4 different classes, and many inhibitors have been developed which can inhibit various HDAC isoforms [12]. Mocetinostat is a class I and IV selective HDAC inhibitor which has shown potent and selective antiproliferative effects in various human cancer cells preclinically [13]. Clinical trials show that mocetinostat is well-tolerated, with favorable pharmacokinetics and pharmacodynamics and promising antitumor activity in several hematological diseases [14]. Combining mocetinostat with other antitumor agents for the treatment of solid tumors is an additional promising approach [15].

In this study we examined a combination of mocetinostat and gemcitabine in the treatment of LMS. In addition to elucidating the *in vitro* anticancer effects, we used an established LMS xenograft model to study the combination effects of gemcitabine and mocetinostat *in vivo*. Our results demonstrate synergistic anti-LMS effects of combined gemcitabine and Mocetinostat, an approach that warrants further study.

## Materials and methods

### Cell lines and reagents

The human LMS cell lines SKLMS1 (ATCC) and LMS1 (Dominique Broccoli, Mercer University, Savannah, GA) was used in this study. LMS cell strains Leio-012 and Leio-196A were developed at the University of Texas MD Anderson Cancer Center/MDACC (Houston, TX) under IRB approval from MDACC and with patient written informed consent. LMS-117 was developed in the Sarcoma Research Lab (The Ohio State University, Columbus, OH) under IRB approval from The Ohio State University and with patient written informed consent. LMS1 was developed in Dr. Dominique Broccoli’s lab (Mercer University, Savannah, GA) and acquired via collaboration; no identifying patient information was provided when acquiring LMS1. To establish LMS cell strains, patient tumor tissue was acquired at surgery and tumor tissue was digested using 3% collagenase Type I, 0.02% DNAse I Type II, and 1.5 mg/mL hyaluronidase. The digested tumor was strained and centrifuged at 1500 RPM for 5 minutes at room temperature, the cell pellet was washed once with sterile PBS and centrifuged again. The cell pellet was resuspended and maintained in DMEM 1X media supplemented with 10% FBS and Primocin. Short Tandem Repeat (STR) analysis was used to confirm the cell origin to the corresponding patient tumor (S1 Table). Control cell lines, human aortic smooth muscle cells (HASMC) and human colonic smooth muscle cells (HCSMC) were used and maintained per supplier protocol (ScienCell). Antibodies used for Western blot analysis: acetylated histone 3 (EMD Millipore), acetylated histone 4 (EMD Millipore), acetylated tubulin (Sigma), RRM1 (Abcam), RRM2 (Abcam), hENT1 (Santa Cruz Biotechnology), β-actin (Santa Cruz Biotechnology), GAPDH (Santa Cruz Biotechnology).

### Cell growth assays

Cell growth conducted via MTS analysis (CellTiter96 Aqueous Non-Radioactive Cell Proliferation Assay kit, Promega) per manufacturer protocol. Cells were plated at 5,000 cells per well and allowed to adhere overnight. Growth rates were assessed 96 hr after treatment with DMSO (control), or 0.1, 0.5, 1 μM of mocetinostat and absorbances measured at 490 nm wavelength. Clonogenic analysis was conducted by plating 400 cells per well. On the following day, cells were treated with DMSO or mocetinostat for 24 hr. Cells were grown for 10 days. On day 10, colonies were stained with 0.5% crystal violet solution for 30 min at RT. Crystal violet solution was removed, wells washed 5x with deionized H2O, stained colonies were scanned and counted. Drug synergy studies were conducted using MTS and analysis was conducted using Compusyn software [16].

### Apoptosis assays

Apoptosis was determined by quantifying cleaved caspase 3 and cleaved caspase 7 activity using Cell Event Caspase 3/7 Green Detection Reagent (Life Technologies) analyzed in the IncuCyte Zoom (Essen BioScience). Cells were plated at 2000 cells per well and treated with the Caspase 3/7 reagent together with DMSO or varying concentrations of mocetinostat for 96 hr. Endpoint analysis was performed in each well using Vybrant DyeCycle Green Stain (Life Technologies) in complete DMEM media.

### *In vivo* experiment

Six week old female SCID mice (n = 40) weighing approximately (AVG±SD) 20±0.9 grams (Taconic Biosciences, Hudson, NY) were injected s.c. with SKLMS1 (1 x 10^6^) into the right flank. Once tumors reached approximately 0.5 cm, mice were randomized into four treatment arms (n = 9 per treatment arm) and treatment was initiated: Vehicle (PEG400/0.2 N HCl), mocetinostat (50 mg/kg PO QD) (Mirati Therapeutics, Inc.), gemcitabine (20 mg/kg, i.p. BID) (Selleck Chemicals), mocetinostat combined with gemcitabine. Mice in the ‘mocetinostat combined with gemcitabine’ treatment arm were given mocetinostat 24 hr prior to combining with gemcitabine. Four mice were excluded from experimentation due to low tumor take. Drug doses were used per manufacture recommendation. Mice were monitored for well being, weighed, and tumors were measured twice weekly. Animals appeared to be in good health with no significant weight loss or death observed during the course of the experiment. Ulceration occurred in some mice at the tumor site nearing the 1.5 cm endpoint; mice were provided with buprenorphine (0.05–0.1 mg/kg) as analgesic. Mice were humanely euthanized (euthanized by CO2 followed by cervical dislocation to ensure death according to IACUC guidelines) once tumors in control mice grew to approximately 1.5 cm. Final tumor volumes and weights were measured. All mice were maintained under barrier conditions at a temperature of 72°F±4°F and 12∶12 hr light:dark cycle. Mice (n = 5/cage) were housed in 194×178×397 mm cages (NexGen caging, Allentown Inc, Allentown, NJ) given feed (Harlan Teklad Irradiated diet 7912, Envigo, Huntingdon, UK) and water *ad libitum*. Environmental enrichment included bedding (1/4” corn cob) and autoclaved nesting material. All procedures were conducted under The Ohio State University’s Institutional Animal Care and Use Committee (IACUC) approval (number 2014A00000085) and in accordance with The Ohio State University’s Animal Welfare Assurance (number A3261-01). All sections of this report adhere to the ARRIVE Guidelines for reporting animal research [17]. A completed ARRIVE guidelines checklist is included in S1 Checklist.

### Statistical analysis

Statistical analysis was performed using GraphPad Prism6. Mean ± SEM was calculated for all *in vitro* and *in vivo* assays using ANOVA. Significance was set at * (*p*≤0.05), ** (*p*≤0.01), *** (*p*≤0.001), **** (*p*≤0.0001). Median values and confidence interval (95%) for all data are located in the S2 Table.

## Results

### Mocetinostat abrogates cell growth and induces apoptosis in LMS cells

**Fig 1:**
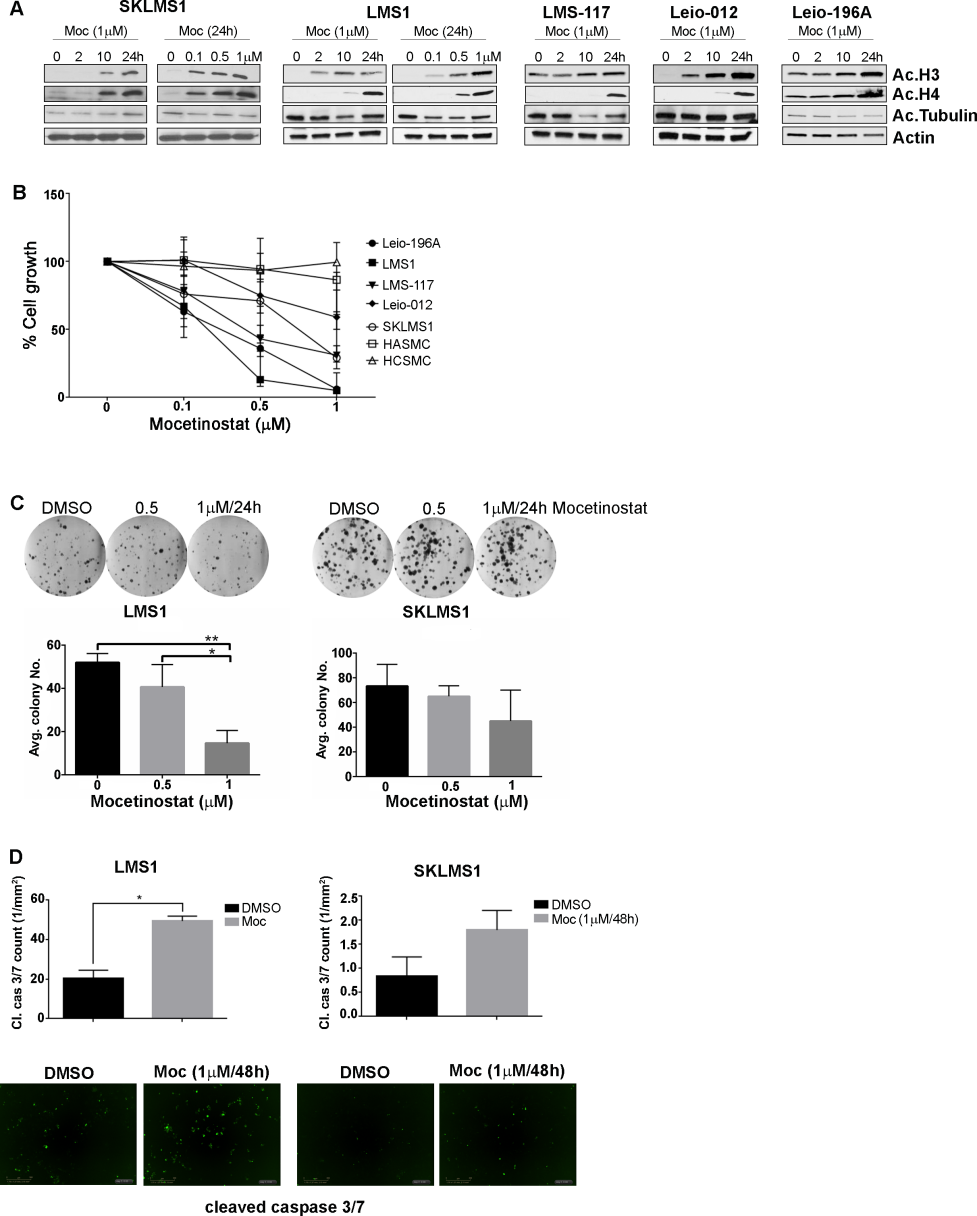
Mocetinostat inhibits LMS cell growth and induces apoptosis. A, Mocetinostat increased acetylated histone 3 and 4 in a time- and dose-dependent manner in LMS cells. Mocetinostat did not increase acetylated tubulin expression. B, Mocetinostat-induced growth inhibition was determined using MTS assays. C, Colony formation assays recapitulate the sensitivity and tolerant dichotomy between LMS1 and SKLMS1 to mocetinostat treatment. D, Mocetinostat induced a significant increase in cleaved caspase 3/7 in LMS1 cells and a modest increase in SKLMS1 cells.

The class I HDAC inhibitor, mocetinostat was used in all experiments. We previously demonstrated the efficacy of the pan-HDAC inhibitor abexinostat/PCI-24781 on a subset of STS cell lines, including the leiomyosarcoma cell line SKLMS1 [18, 19]. Pan-HDAC inhibitors, per abexinostat, demonstrate a high affinity to class I HDAC isoforms; e.g., HDAC1 and HDAC2 [20]. Pan-HDAC inhibitors yield numerous undesirable side effects, whereas the inhibition of various HDAC isoforms results in improved therapeutic windows and less toxicity [21], thus providing an impetus to target a smaller set, i.e., class I HDACs in LMS. Mocetinostat increased LMS cell line/strain histone 3 and 4 acetylation in a dose- and time-depended manner (Fig 1A). Tubulin acetylation, a HDAC6 substrate, remained unacetylated upon treatment with mocetinostat, demonstrating the compound’s affinity to class I *vs* class II HDAC substrates. The effect of mocetinostat on LMS cell growth was tested. Mocetinostat abrogated cell growth in a time- and dose-dependent manner (Fig 1B and S3 Table). LMS1 and Leio-196A demonstrated sensitivity to mocetinostat followed by LMS-117; SKLMS1 and Leio-012 exhibited the highest tolerance among the LMS cells. Mocetinostat had a modest impact on normal cells (HASMC and HCSMC). Mocetinostat significantly reduced LMS clonogenic potential in LMS1 and SKLMS1 cells (Fig 1C). Again, LMS1 exhibited sensitivity whereas SKLMS1 was more tolerant to mocetinostat anti-cancer effects.

To further assess the impact of mocetinostat on LMS cell proliferation, we evaluated the compound’s effect on apoptosis. Cleaved caspase 3/7 activity in LMS cells treated with mocetinostat was measured using the IncuCyte Zoom system. A similar trend in drug sensitivity was observed in LMS cells in response to caspase 3/7 activity (Fig 1D); LMS1 displaying a significant increase in cleaved caspase 3/7 and SKLMS1 displaying a modest cleaved caspase 3/7 increase in response to mocetinostat.

### Mocetinostat combined with gemcitabine exhibits a synergistic anti-LMS effect in vitro

**Fig 2:**
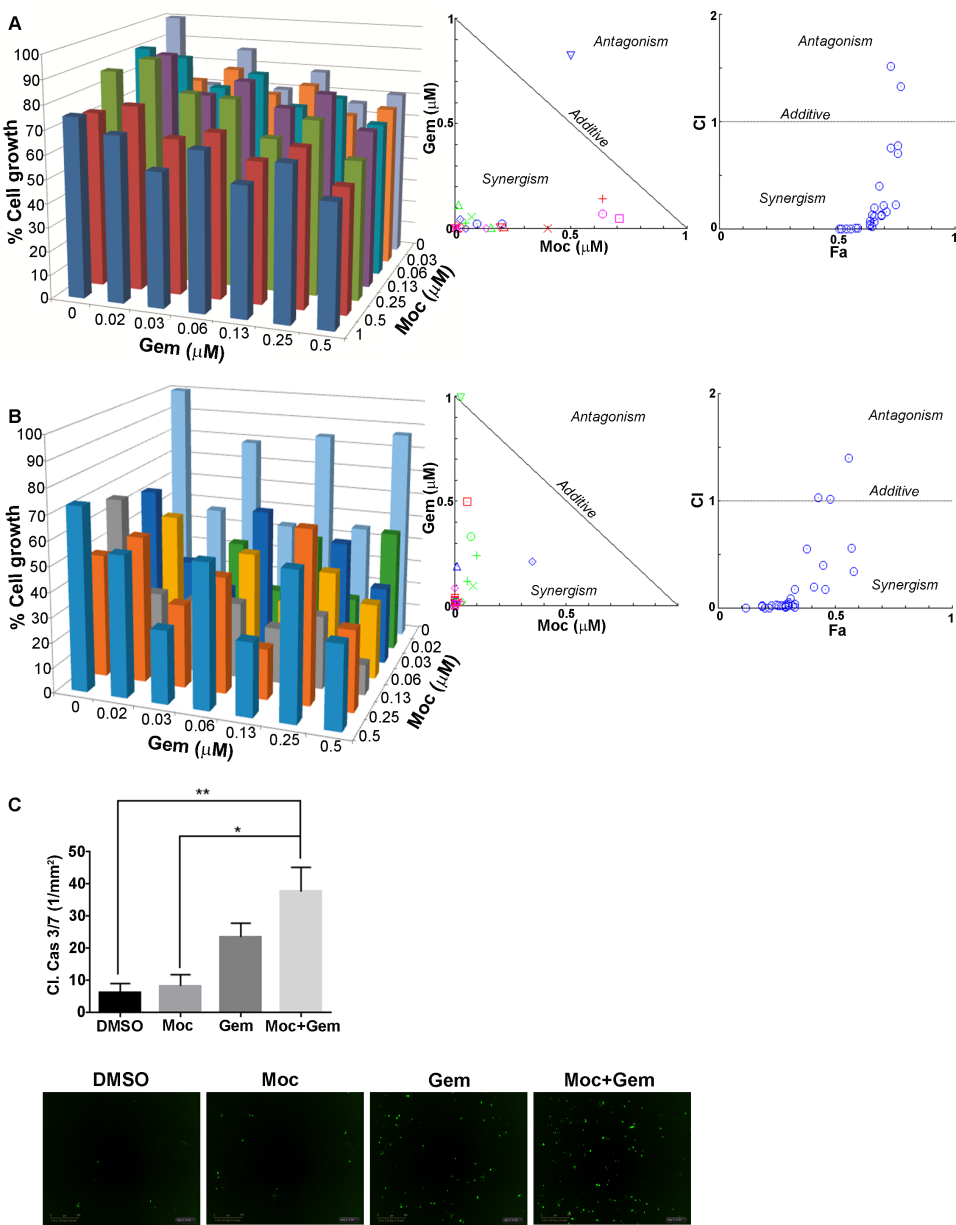
Mocetinostat synergizes with gemcitabine in LMS cells *in vitro*. A and B, MTS assays demonstrating synergistic combination of mocetinostat and gemcitabine in SKLMS1 and LMS cells, respectively (cells were pretreated with mocetinostat prior to combining with gemcitabine). C, Mocetinostat combined with gemcitabine enhances caspase 3/7 positive SKLMS1 cells. Combination index (CI) values of the drug combinations were calculated using Compusyn software. Synergy, additivity, and antagonism are defined as CI < 1, CI = 1, and CI > 1, respectively.

Previously, we identified superior anti-STS effects when combining pan-HDAC inhibition (abexinostat) with doxorubicin or cisplatin [18] *in vitro* and *in vivo*. In this study we evaluated the efficacy of mocetinostat combined with gemcitabine. SKLMS1 and LMS1 were used as representative cell lines that were tolerant/sensitive in response to mocetinostat. Cells were pretreated with mocetinostat 24 hr prior to combining with gemcitabine. The combination of mocetinostat and gemcitabine in this dosage sequence exhibited a synergistic effect in both cell lines (Fig 2A and 2B). No synergy was identified when the cells were pretreated with gemcitabine prior to combining with mocetinostat or when both drugs were administered concurrently (data not shown). The combination effects on apoptosis were evaluated next. SKLMS1 cells were pretreated with mocetinostat then combined with gemcitabine and analyzed for caspase 3/7 activity using the Incucyte Zoom. The data demonstrates a significant pro-apoptotic effect in cells treated with the combination versus either drug alone (Fig 2C). These *in vitro* findings lead us to test this combination *in vivo*.

### Mocetinostat combined with gemcitabine versus either drug alone demonstrates a superior anti-LMS effect *in vivo*

**Fig 3:**
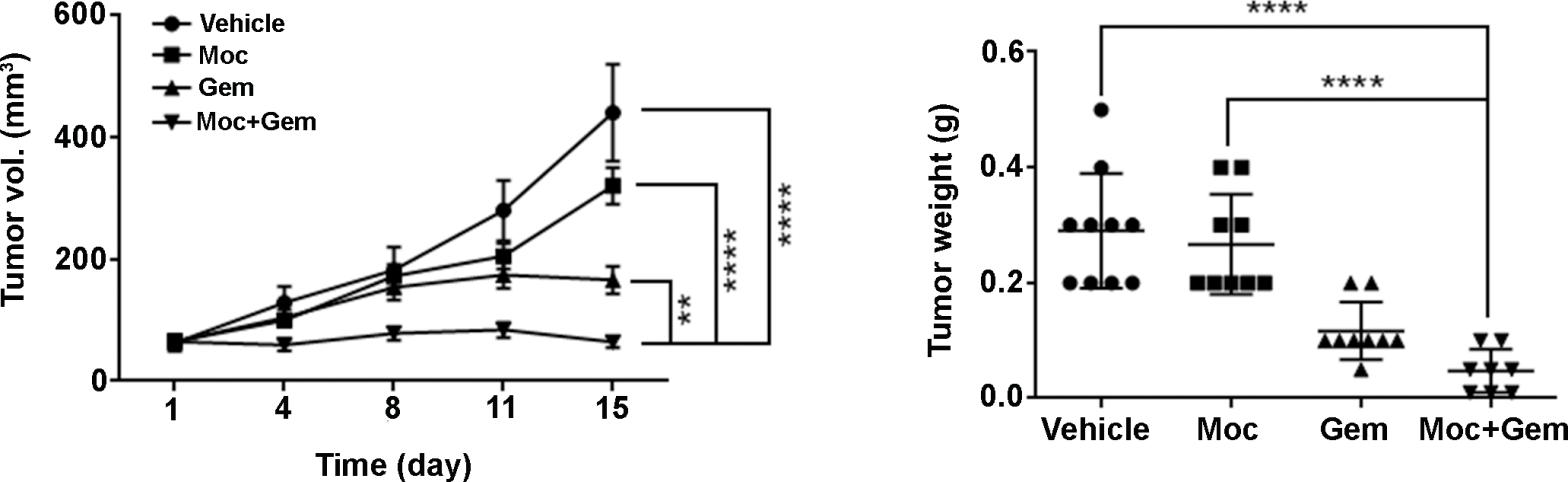
Mocetinostat combined with gemcitabine exhibits significant anti-LMS effect *in vivo*. Mocetinostat combined with gemcitabine significantly reduced tumor growth and tumor weight.

SKLMS1 xenografts were used for *in vivo* testing of mocetinostat combined with gemcitabine in that LMS1 cells failed to grow as xenografts in SCID mice. Once tumors reached approximately 100 mm^3^, mocetinostat was administered i.p. daily at a dose of 50 mg/kg to mice in the mocetinostat alone and mocetinostat plus gemcitabine groups. The treatments were well tolerated without significant weight loss. Treatment with mocetinostat alone did not significantly affect SKLMS1 tumor growth, whereas gemcitabine alone induced significant tumor growth inhibition (*p* ≤ 0.0001). Mocetinostat combined with gemcitabine significantly inhibited SKLMS1 tumor growth compared to either treatment alone (Fig 3; *p* ≤ 0.0001). The average tumor weights at the end of the study were: 0.29 g ±0.03 for vehicle, 0.27 g ±0.03 for mocetinostat alone, 0.12 g ±0.02 for gemcitabine alone, and 0.05 g ± 0.01 for the combination (Fig 3). Mocetinostat alone modestly reduced tumor weight, while gemcitabine alone (*p* ≤ 0.0001) and the drug combination significantly (*p* ≤ 0.0001) reduced SKLMS1 tumor weight compared to vehicle.

### Mocetinostat reduces gemcitabine-resistance associated target expression *in vitro* and *in vivo*

**Fig 4:**
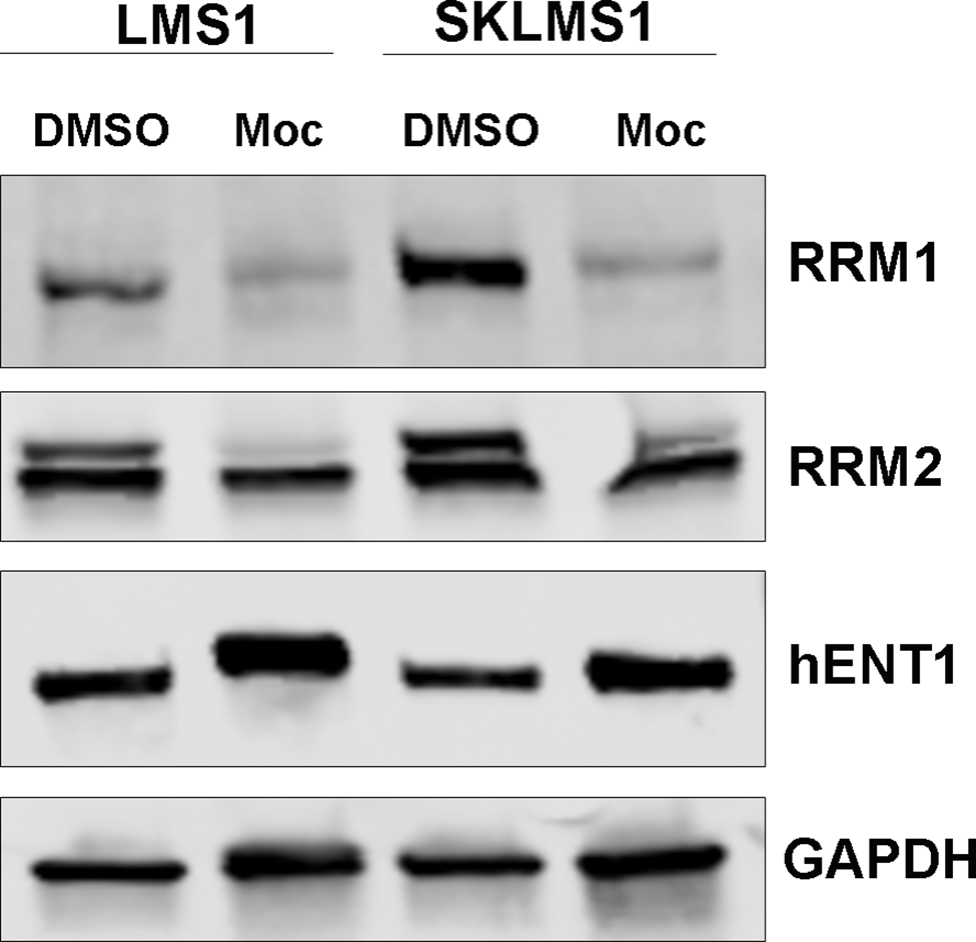
Mocetinostat regulates gemcitabine-resistant markers expression. Mocetinostat reduces RRM1 and RRM2, and increases hENT1 expression in LMS cell lines.

To identify potential mechanisms of mocetinostat-induced sensitization to gemcitabine, LMS cells were treated with mocetinostat and immunoblotted for the key gemcitabine-resistant targets, RRM1, RRM2, and hENT1 [22, 23], demonstrating mocetinostat-induced down-regulation of RRM1, RRM2, and an increase in hENT1 protein expression in LMS cells (Fig 4). Mocetinostat-induced regulation of these proteins may contribute to the observed synergistic sensitization to gemcitabine *in vitro* and *in vivo*, and this direction is being evaluated by us at this time.

## Discussion

Our investigations demonstrate anti-LMS synergy using the combination of mocetinostat and gemcitabine. Our preclinical data explores a novel therapeutic approach for the treatment of LMS. The role of gemcitabine in sarcoma is limited. Initially, gemcitabine was shown to have benefit for the treatment of chemo-resistant osteosarcoma [24], leading the way for various clinical trials in sarcomas of different histologies. The trials demonstrate uterine LMS (ULMS) to respond best to gemcitabine compared to non-uterine LMS and STS of different histology [8]. Recently, markers associated with gemcitabine resistance were shown to be upregulated in a cohort of ULMS patient samples [25]. Studies focusing on the role of HDAC inhibition in STS warrants further investigation, especially in the context of LMS [18, 26, 27].

The efficacy of HDAC inhibition combined with gemcitabine has been previously described in various tumor models [28–41]. Extensive examination of gemcitabine resistance in pancreatic cancer has yielded strategies to overcome gemcitabine resistance using rational combination therapies, including several classes of HDAC inhibitor compounds. Similar to our study, Sung et al. demonstrated the synergistic efficacy of mocetinostat in combination with gemcitabine in human pancreatic cancer cells [42]. Gemcitabine resistant cells have been shown to be sensitive to HDAC inhibition [43]. Recently, Lee et al., demonstrated that the novel HDAC inhibitor, CG200745, reduced the expression of multidrug-resistant protein MRP4 in pancreatic cells, suggesting a potential synergistic mechanism of HDAC inhibition combined with gemcitabine [44].

Pan-HDAC inhibitors exhibit side effects prompting the development of isoform-specific HDAC inhibitors, with the aim of reducing toxicities while enhancing the therapeutic window. Gong et al. demonstrated the effective combination of sirtuin 1 (SIRT1) inhibition and gemcitabine in pancreatic carcinoma. Sirtuin 1 is one of seven NAD-dependent deacetylase isoforms [45], suggesting that the combination of HDAC/SIRT isoform-specific inhibition and gemcitabine may warrant further investigation.

Epigenetic regulation of acquired gemcitabine resistance has been described [46–49]. HDAC inhibition may be crucial to help establish potential epigenetic markers of LMS gemcitabine resistance that can be targeted for therapy. Essential proteins that have a role in gemcitabine resistance include RRM1, RRM2, and hENT1. We have demonstrated mocetinostat-induced regulation of these proteins, suggesting a potential mechanism of mocetinostat-induced sensitivity to gemcitabine. In LMS, RRM1 is more highly expressed in uterine LMS versus extrauterine LMS patients [25], a possible biological dichotomy relevant to therapeutic intervention. The roles of RRM2 and hENT1 in the context of gemcitabine resistance in leiomyosarcoma have yet to be identified, and are currently under study in our laboratory.

Our work expands the knowledge base needed to identify potentially novel therapeutic options for the treatment of leiomyosarcoma, especially in the context of chemotherapeutic resistance. This work also identifies novel LMS cell lines; there is a major lack of available LMS cell lines at this time. This study presents a platform to further study the mechanistic role of HDAC inhibition and gemcitabine resistance in LMS. Accordingly, we currently investigating isoform-specific HDAC inhibition combined with gemcitabine to determine the utility of specific HDAC isoforms in anti-LMS therapy, including their potential synergism with gemcitabine as per above. These studies also utilize several LMS patient-derived xenograft (PDX) models we have recently developed as a preclinical bridge to future clinical trials to evaluate these severely needed and promising new approach to LMS patent therapeutics.

## Supporting information

S1 Checklist The ARRIVE guidelines checklist. (PDF)

S1 Table STR analysis of LMS cell lines compared to their corresponding patient tumor. (XLSX)

S2 Table Median values and confidence intervals (95%). (XLSX)

S3 Table EC50 of mocetinostat on cell lines. (XLSX)
